# Overexpression of miR-671-3p alleviates postmenopausal osteoporosis by targeting *GREM2* to activate *BMP2/SMAD* signaling pathway

**DOI:** 10.1186/s41065-025-00467-8

**Published:** 2025-06-11

**Authors:** Yanlin Liang, Changqing Gu, Peng Wang, Changwen Gu, Hongwei Ma, Shujun Ren

**Affiliations:** 1 Orthopedics Department, Ningxia Chinese Medicine Research Center, No. 114, Beijing West Road, Xixia District, Yinchuan, 750021 China; 2 Department of Orthopedic Trauma, Yinchuan Guolong Orthopedic Hospital, Yinchuan, 750001 China; 3 Orthopedics Department, First Affiliated Hospital, Heilongjiang University of Chinese Medicine, No.26, Heping Road, Xiangfang District, Harbin, 150036 China

**Keywords:** PMOP, Osteogenic differentiation, Microgravity, miR-671-3p, *GREM2*

## Abstract

**Background:**

Increased fracture risk is linked to postmenopausal osteoporosis (PMOP), and elucidating the function of microRNAs (miRNAs) in this condition is vital for identifying individuals at high risk of fractures. This research focused on exploring the function and mechanism of miR-671-3p in PMOP.

**Methods:**

Using qRT-PCR, we measured the expression levels of miR-671-3p in the serum of PMOP patients and evaluated its predictive capacity for osteoporosis occurrence through Receiver Operating Characteristic (ROC) analysis. An in vitro model of MC3T3-E1 osteoblasts cultured under simulated microgravity (MG) was established to mimic the osteoporosis-related bone loss microenvironment. It was used to investigate miR-671-3p’s regulatory effects on cell proliferation (CCK-8 assay), apoptosis (Annexin V/PI staining), and osteogenic differentiation (ALP activity and osteogenic marker mRNA levels). Dual luciferase reporter gene assays and RNA immunoprecipitation (RIP) experiments were performed to validate the interaction between miR-671-3p and *GREM2*.

**Results:**

miR-671-3p expression was reduced in PMOP patients and in MG-exposed MC3T3-E1 cells. miR-671-3p exhibited strong predictive power for early detection of PMOP. When miR-671-3p was overexpressed, it enhanced osteogenic differentiation and suppressed apoptosis in MC3T3-E1 cells. *GREM2* was pinpointed as a target of miR-671-3p, which inhibited osteogenic differentiation in MC3T3-E1 cells and accelerated MG-induced apoptosis. By inhibiting *GREM2* expression, overexpression of miR-671-3p activated the *BMP2/SMAD* signaling pathway.

**Conclusion:**

Reduced miR-671-3p expression may signal the presence of PMOP. By targeting *GREM2* to activate the *BMP2/SMAD* pathway, miR-671-3p may stimulate osteogenic differentiation, foster bone formation, and prevent the onset of osteoporosis.

**Supplementary Information:**

The online version contains supplementary material available at 10.1186/s41065-025-00467-8.

## Introduction

Postmenopausal osteoporosis (PMOP) is a metabolic disorder that significantly impacts the quality of life for middle-aged and elderly women. It arises due to ovarian dysfunction and a sharp decline in estrogen levels, which disrupts the bone tissue’s microarchitecture, resulting in decreased bone mass and mineral density [1]. This, in turn, enhances bone fragility and significantly raises the risk of fractures. With the rapid aging of the global population, PMOP has become a pressing public health concern, imposing heavy burdens on both society and individuals. In the development of osteoporosis, osteoblasts are vital as they are pivotal cells for bone formation [2]. A lack of estrogen not only impacts osteoblasts differentiation and activity directly but may also indirectly regulate their functions via complex signaling networks [3]. Hence, a thorough investigation into the molecular mechanisms underlying osteoblast activity in individuals with PMOP is crucial for identifying new predictive markers and therapeutic targets for this condition.

MicroRNAs (miRNAs), though not directly involved in protein coding, effectively regulate gene expression at the post-transcriptional level by inhibiting the expression of target genes or promoting the degradation of target mRNAs through complementary pairing with their 3’ untranslated regions (3’UTRs) [4]. As key regulators of gene expression, miRNAs have shown great potential in studying the pathogenesis of various diseases. Recent advancements in high-throughput sequencing technologies have revealed abnormal miRNAs expression was closely linked to the onset and progression of PMOP [5]. They play a crucial role in regulating the differentiation and function of osteoblasts and osteoclasts, maintaining a delicate balance between bone formation and resorption [6]. For example, studies have demonstrated a strong association between the abnormal expression of miR-4739 and PMOP progression [7], while miR-33a-3p, by targeting insulin-like growth factor 2 (*IGF2*) to influence osteogenic differentiation, holds promise as a biomarker and therapeutic target for PMOP [8].

Notably, the potential role of miR-671-3p in skeletal system-related diseases has garnered attention. Ntoumou E et al. found that miR-671-3p could serve as a potential biomarker for metabolic osteoarthritis [9]. It has been discovered to play a pivotal role in modulating inflammatory responses and cellular functions in chondrocytes [10]. Several studies have also indicated that miR-671-3p has emerged as a significant factor in numerous diseases, influencing a range of cellular biological processes. For instance, in non-small cell lung cancer, miR-671-3p is downregulated and inhibits cancer cell proliferation and metastasis by directly targeting *CCND2* [11]. It also plays a crucial regulatory role in the proliferation and differentiation of intramuscular adipocytes [12]. Additionally, miR-671-3p promotes glioma cell proliferation and migration by targeting *CKAP4* [13]. It is aberrantly expressed in endometrial cancer and may be used as a diagnostic biomarker and it is closely linked to cell carcinogenesis [14]. However, despite the proven regulatory role of miR-671-3p in bone-related diseases and cellular biological processes, its specific function in PMOP and its involvement in osteoblasts activity remain unclear and warrant further investigation.

Therefore, this study is dedicated to exploring the expression profile of miR-671-3p in PMOP and its intricate functions and mechanisms in regulating osteoblast activity, aiming to provide new insights into the prevention and intervention of PMOP.

## Materials and methods

### Study participants

Between 2019 and 2021, First Affiliated Hospital, Heilongjiang University of Chinese Medicine hospitals were selected to recruit postmenopausal female participants for the study. The research was conducted under the approval and oversight of the First Affiliated Hospital, Heilongjiang University of Chinese Medicine Ethics Committee, ensuring that all involved personnel were informed, voluntarily participated, and had signed consent forms. The participants were divided into two groups based on their osteoporosis status [15], comprising 85 patients in the PMOP group and 83 patients without osteoporosis. The sample size was initially determined by clinical feasibility and prior research experience, and further validated using G*Power statistical software with parameters set as α = 0.05, power = 0.8, and effect size = 0.5. The analysis indicated a minimum required sample size of 51 participants per group, which was met by the enrolled population to ensure scientific rigor. Inclusion criteria for participants were as follows: (1) no prior use of medications affecting bone metabolism (such as bisphosphonates, Denosumab, or estrogens); (2) at least 1 year of menopause; (3) absence of other chronic malignant or congenital diseases; (4) no history of metabolic bone diseases or secondary osteoporosis; and (5) complete clinical data.

All participating clinical data and blood samples were obtained, with serum separated and frozen for storage.

### Cell culture and cell transfection

The MC3T3-E1 pre-osteoblast cells were sourced from Geneo Biotechnology Co., Ltd. (Guangzhou, China). Frozen cells were thawed via water bath and cultured in DMEM complete medium, which contained 100 mg/L streptomycin, 100 mg/L penicillin, and 10% fetal bovine serum (FBS). This was done in a 5% CO_2_, 37℃ steady temperature incubator. Once the cells reached about 80% confluence, they were trypsinized and the digestion process was halted with complete medium. The cells were then passaged at a 1:3 ratio. Logarithmically growing cells were chosen for follow-up experiments. The MC3T3-E1 cells of the fourth generation were cultured under standard conditions, and transfection was initiated once the cells reached 80% confluence. The miR-671-3p mimic plasmid (5’-UAGGAUACCUGGGUGGGGUC-3’) was utilized to overexpress miR-671-3p, with the miR-NC plasmid (5’-UUCUCCGAACGUGUCACGUTT-3’) serving as a non-targeting negative control. The plasmids were synthesized by GenePharma (Shanghai, China). According to the instructions provided by Lipofectamine™ 3000 (Thermo Fisher Scientific Co., Ltd., China), 1 µg of transfection plasmid was mixed with 2 µL of Lipofectamine 3000 solution. Subsequently, this mixture was introduced into the MC3T3-E1 cells.

### Simulated microgravity

A two-dimensional rotational system developed by the China Astronaut Research and Training Center in Beijing has been used to establish microgravity (MG) -simulated environments for the cellular studies. This methodology, validated through prior research, effectively recreates MG-induced osteoporotic microenvironments at the cellular level [16, 17]. Cells were plated at a density of 1 × 10^5^ cells per milliliter in a rotating culture system. Subsequently, 6 mL of microcarrier solution (concentration: 5 mg/mL) were introduced, and bubbles were meticulously removed using a pipette. The cultures were maintained at a consistent rotational speed of 20 revolutions per minute for 72 h. Whenever air bubbles were observed, they were promptly eliminated to prevent any adverse effects of fluid shear force on the cells during the cultivation period. Meanwhile, the control group underwent standard cultivation in cell culture flasks.

### Osteogenic differentiation induction

For the experiment, cells that had undergone successful transfection were chosen for induction of osteoblast differentiation. These cells were then exposed to an osteogenic differentiation medium containing 10% FBS, 50 µg/mL ascorbic acid, 5mM β-glycerophosphate sodium, and 2mM glutamine. Culturing conditions remained as previously detailed, with both osteogenic induction and simulated microgravity treatment being applied concurrently.

### Cell viability

Cell viability was assessed using the CCK-8 kit from Solaibao Biotechnology Co., LTD (Beijing, China). Cells successfully induced differentiated under MG treatment comprised the experimental group, while those without MG treatment served as the control group. Both groups were adjusted to a uniform cell suspension concentration of 10^3^ cells/mL and seeded into 96-well plates. After 48 h, Each well was seeded with 10 µL CCK-8 solution and incubated for a further 2 h. Absorbance at 450 nm was then measured for each group using a fully automated enzyme marker.

### Cell apoptosis

Apoptosis rates were determined using the Apoptosis Detection Kit (Biyuntian Biotechnology Co., Ltd., Shanghai, China), analyzed by flow cytometry. Briefly, cell suspension were stained with 5 µL of Annexin V and 10 µL of PI in the dark at room temperature for 10 min. Subsequently, apoptotic cells were quantified using a flow cytometer (Attune NxT; Thermo Fisher Scientific) and ModFiLT software (v4.1, Verity Software House).

### Osteoblast ALP activity assay

To measure osteoblast ALP activity, cell supernatants from each group were collected, centrifuged at 1,200 rpm for 10 min at 4 °C, and then processed according to the ALP kit instructions (Solaibao Biotechnology Co., LTD, Beijing, China). After gentle shaking, the absorbance of each group was measured at a wavelength of 520 nm using a microplate reader.

### Molecular relative expression assay

Serum and cells were transferred to centrifuge tubes and total RNA was extracted using TRIzol reagent (Jinheng Pharmaceutical Technology Co., Ltd., Guangzhou, China). RNA concentration (> 200 ng/µL) was measured with NanoDrop 2000, and RNA integrity was detected by agarose gel electrophoresis. The RNA obtained was reverse transcribed to synthesise cDNAs (Zhenjie Biotechnology Co., Ltd., Shanghai, China). The cDNAs were used as templates to detect the expression of Gremlin-2 (*GREM2*), runt-related transcription factor 2 (*RUNX2*), osteocalcin (*OCN*), ccollagen, type I, alpha 1 (*COL1A1*) mRNA and miR-671-3p by real-time fluorescence quantitative PCR (SYBR Green Realtime PCR Master Mix, Crestview Biotechnology Co Ltd, Qingdao, China). Primers were purchased from Shanghai Hop Sing Biotechnology Co. *U6* and *GAPDH* were used as internal reference genes and calculated with the help of the 2^−ΔΔCt^ formula.

### Dual luciferase gene assay report

Initial screening of potential miR-671-3p target genes was performed through intersection analysis of prediction results from the TargetScan (http://www.targetscan.org/vert_72/) and StarBase (https://rnasysu.com/encori/panCancer.php) databases. Priority was given to genes implicated in bone-related diseases based on previous research findings, leading to the selection of *GREM2* for further investigation. Depending on the presence or absence of the binding site, the wild-type plasmid WT-*GREM2* and the mutant plasmid MT-*GREM2* were constructed. The two plasmids were transfected with miR-671-3p mimic, inhibitor and control mixture into MC3T3-E1 cells via LipofectamineTM 3000. After 24 h of transfection, the two plasmids were tested by dual-luciferase assay (Solaibao Biotechnology Co. Beijing, China) to detect luciferase activity.

### RNA Immunoprecipitation (RIP) assays

RIP assays were performed using the EZ-Magna RIP Kit (Millipore, USA) to investigate miR-671-3p-*GREM2* interactions within the RNA-induced silencing complex (RISC). Briefly, MC3T3-E1 cells were lysed in RIP buffer containing protease inhibitors, and lysates were incubated with Protein A/G magnetic beads pre-conjugated with Argonaute-2 (Ago2) antibody (a RISC core protein critical for miRNA-mediated target binding) or IgG control (5 µg per reaction). Following stringent washes to eliminate non-specific associations, RNAs co-precipitated with Ago2-containing RISC complexes were isolated and analyzed by qRT-PCR to assess miR-671-3p-dependent target enrichment.

### Protein expression assay

A Western blot assay was conducted to examine the expression levels of *GREM2*, bone morphogenetic protein 2 (*BMP2*), phosphorylated Drosophila Mothers Against Decapentaplegic Protein 1 (*P-SMAD1*), *P-SMAD5*. Initially, cells were lysed with RIPA buffer to extract total proteins. Subsequently, 40 micrograms of protein were resolved through electrophoresis on a 10% SDS-PAGE gel, followed by transfer onto a polyvinylidene difluoride (PVDF) membrane. Proteins were detected using antibodies against *GREM2* (1:1000, Proteintech), *BMP2* (1:1000, CST), *p-SMAD1/5* (1:1000, Abcam), and *GAPDH* (1:5000, Proteintech). The membrane was then blocked with 5% skimmed milk for a duration of 2 h. After blocking, specific primary antibodies were applied to the membrane and incubated overnight at 4 °C. The membrane was then washed three times with Tris-Buffered Saline with Tween-20 (TBST). Next, horseradish peroxidase-labeled goat anti-rabbit secondary antibody was added and incubated for 1 h at room temperature. Protein expressionwere detectedusing an Enhanced Chemiluminescence (ECL) system (Bio-Rad), with band intensity quantified through grayscale analysis in ImageJ software. *GAPDH* served as the loading control for normalization, calculated as the ratio of target protein to *GAPDH* band intensity.

### Statistical analysis

At least 5 replicate experiments were performed and data are presented as mean with standard deviation (SD). Statistical comparisons were made using either unpaired Student’s t-test or one-way ANOVA with SPSS (version 23.0) and GraphPad Prism 9. Receiver Operating Characteristic (ROC) curves were used to analyse diagnostic accuracy, while associations were assessed using Pearson’s rank correlation coefficient. Results were considered statistically significant at *P* < 0.05.

## Result

### Clinical significance of miR-671-3p in PMOP patients

**Fig. 1:**
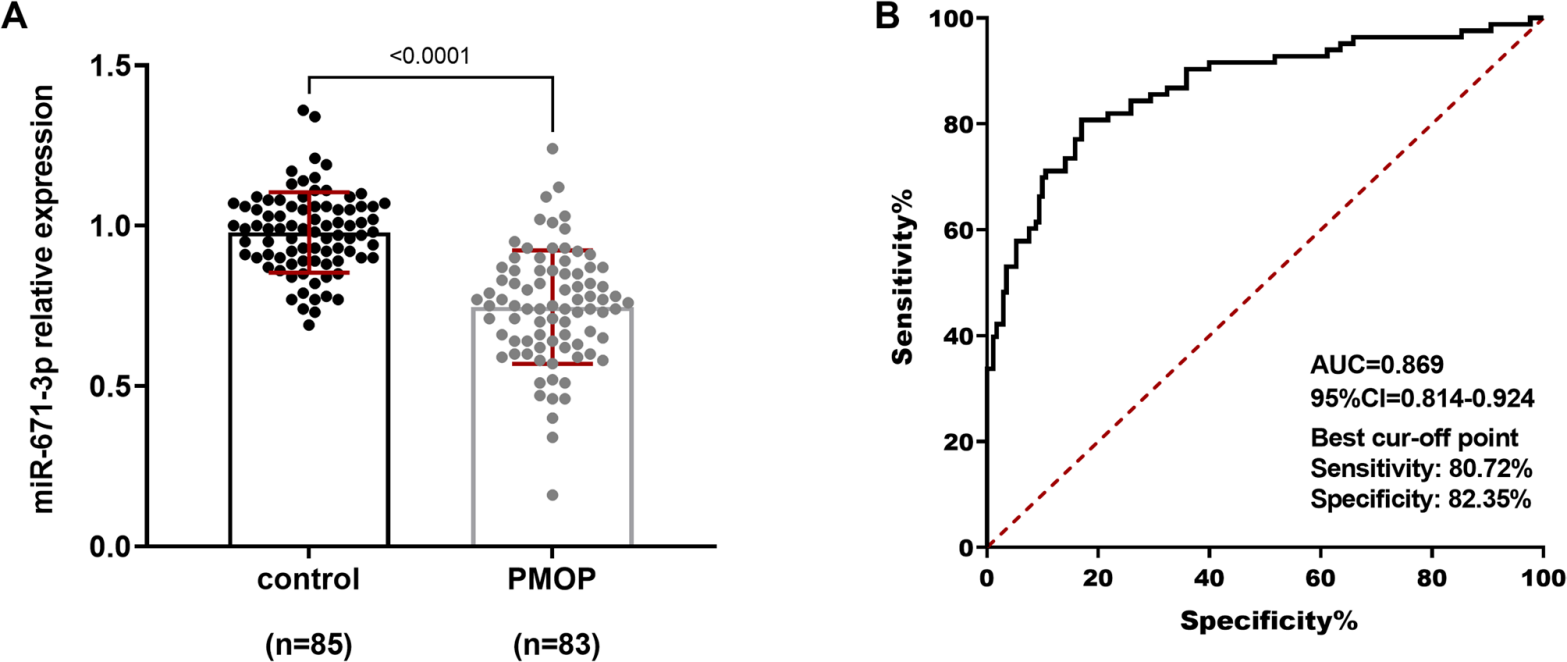
PMOP patients exhibit lower serum levels of miR-671-3p compared to the control group (**A**). ROC analysis suggests that miR-671-3p possesses significant predictive power for assessing postmenopausal female osteoporosis (**B**). Control: Postmenopausal women without osteoporosis (*n* = 83); PMOP: Patients diagnosed with PMOP (*n* = 85). Bar graphs show mean ± SD. Student’s t-test

Bone mineral density (BMD) values at the lumbar spine (LS), femoral neck (FN) and total hip (TH) were remarkably lower in patients with PMOP compared to postmenopausal women without osteoporosis (*P* < 0.05). No significant differences were observed in other clinical parameters (*P* > 0.05), ensuring the scientific rigor of the study (Table 1). qRT-PCR analysis revealed a decreased serum expression level of miR-671-3p in PMOP patients compared to controls (Fig. 1A). ROC analysis demonstrated that miR-671-3p had a strong predictive power for osteoporosis in menopausal women, with an AUC value of 0.869 (Fig. 1B). Furthermore, Pearson correlation analysis showed a significant positive correlation between miR-671-3p and BMD values in all three regions (Table 2, *r* > 0.5, *p* < 0.001).

**Table 1:** Clinical data of the study subjects

Clinical Information	control	PMOP	p
	(n = 85)	(n = 83)	
Age (years)	51.94 ± 4.45	52.65 ± 4.52	0.307
BMI (kg/m^2^)	24.03 ± 1.74	23.77 ± 1.73	0.317
SBP (mmHg)	114.37 ± 15.04	114.27 ± 14.21	0.963
DBP (mmHg)	74.71 ± 8.35	73.91 ± 8.98	0.556
FBG	4.99 ± 0.66	5.01 ± 0.71	0.867
LDL (mmol/L)	2.71 ± 0.38	2.82 ± 0.38	0.085
HDL (mmol/L)	1.54 ± 0.31	1.60 ± 0.30	0.202
TC (mmol/L)	4.55 ± 0.83	4.44 ± 0.70	0.103
TG (mmol/L)	1.22 ± 0.25	1.20 ± 0.27	0.610
LS BMD (g/cm^2^)	0.90 ± 0.03	0.77 ± 0.04	< 0.001
FN BMD (g/cm^2^)	0.72 ± 0.03	0.63 ± 0.04	< 0.001
TH BMD (g/cm^2^)	0.76 ± 0.03	0.67 ± 0.04	< 0.001

BMI (kg/m²): Body Mass Index

SBP (mmHg): Systolic Blood Pressure

DBP (mmHg): Diastolic Blood Pressure

FBG: Fasting Blood Glucose

LDL (mmol/L): Low - Density Lipoprotein Cholesterol

HDL (mmol/L): High - Density Lipoprotein Cholesterol

TC (mmol/L): Total Cholesterol

TG (mmol/L): Triglyceride

LS BMD (g/cm²): Lumbar Spine Bone Mineral Density

FN BMD (g/cm²): Femoral Neck Bone Mineral Density

TH BMD (g/cm²): Total Hip Bone Mineral Density​

**Table 2:** Correlation between miR-671-3p and BMD

Indicators	miR-671-3p	
	r	P value
LS BMD (g/cm2)	0.772	< 0.0001
FN BMD (g/cm2)	0.728	< 0.0001
TH BMD (g/cm2)	0.693	< 0.0001

LS BMD (g/cm^2^): Lumbar Spine Bone Mineral Density; FN BMD (g/cm^2^): Femoral Neck Bone Mineral Density; TH BMD (g/cm^2^): Total Hip Bone Mineral Density

### Impact of miR-671-3p on MC3T3-E1 cell activity in microgravity conditions

**Fig. 2:**
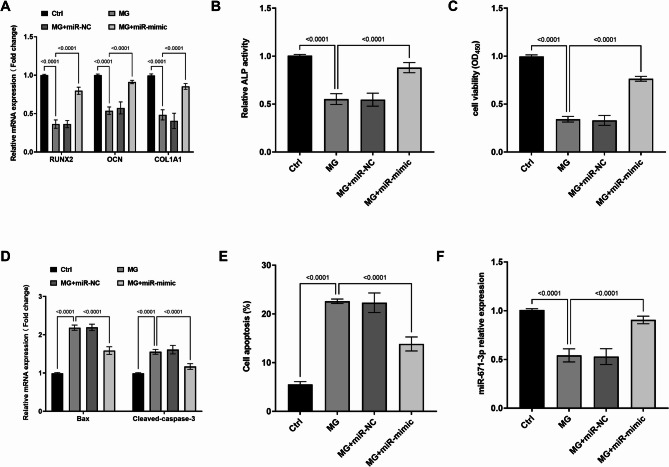
Under MG conditions, the mRNA expressions of osteogenic markers in cells (**A**), the activity of ALP (**B**) and cell viability (**C**) were diminished. The expressions of apoptosis-related mRNAs (**D**) and apoptosis rate (**E**) were enhanced. MG inhibited the expression of miR-671-3p (**F**). Ctrl: Normal culture conditions; MG: Cells treated with simulated microgravity (MG) for 72 h; MG + miR-NC: MG + miR-negative control (NC) transfection; MG + miR-mimic: MG + miR-671-3p mimic transfection. Bar graphs show mean ± SD (*n* = 5). one-way ANOVA with Tukey’s post hoc test

MG treatment mimics mechanical unloading in osteoporosis by suppressing osteogenic differentiation while inducing apoptosis - recapitulating key features of PMOP pathophysiology. Exposure of MC3T3-E1 cells to MG for 72 h resulted in a significant downregulation of osteoblast differentiation markers such as *OCN*, *RUNX2* and *COL1A1* (Fig. 2A). Additionally, the relative activity of ALP was markedly reduced (Fig. 2B), indicating impaired osteoblast differentiation capacity following MG exposure. MC3T3-E1 cell viability was also significantly decreased (Fig. 2C), accompanied by an increase in the expression of apoptosis-related proteins *Bax* and Cleaved-caspase-3 mRNA (Fig. 2D), and a corresponding increase in the apoptosis rate (Fig. 2E). Notably, MG treatment led to a significant reduction in miR-671-3p expression in MC3T3-E1 cells. However, this suppression was effectively reversed by transfection with a miR-671-3p mimic as demonstrated in Fig. 2F. Consequently, the differentiation capacity of MG-treated MC3T3-E1 cells rebounded, accompanied by a marked decrease in apoptosis rate, suggesting a protective role for miR-671-3p in MC3T3-E1 cells under MG conditions.

### Interaction of miR-671-3p with *GREM2*

**Fig. 3:**
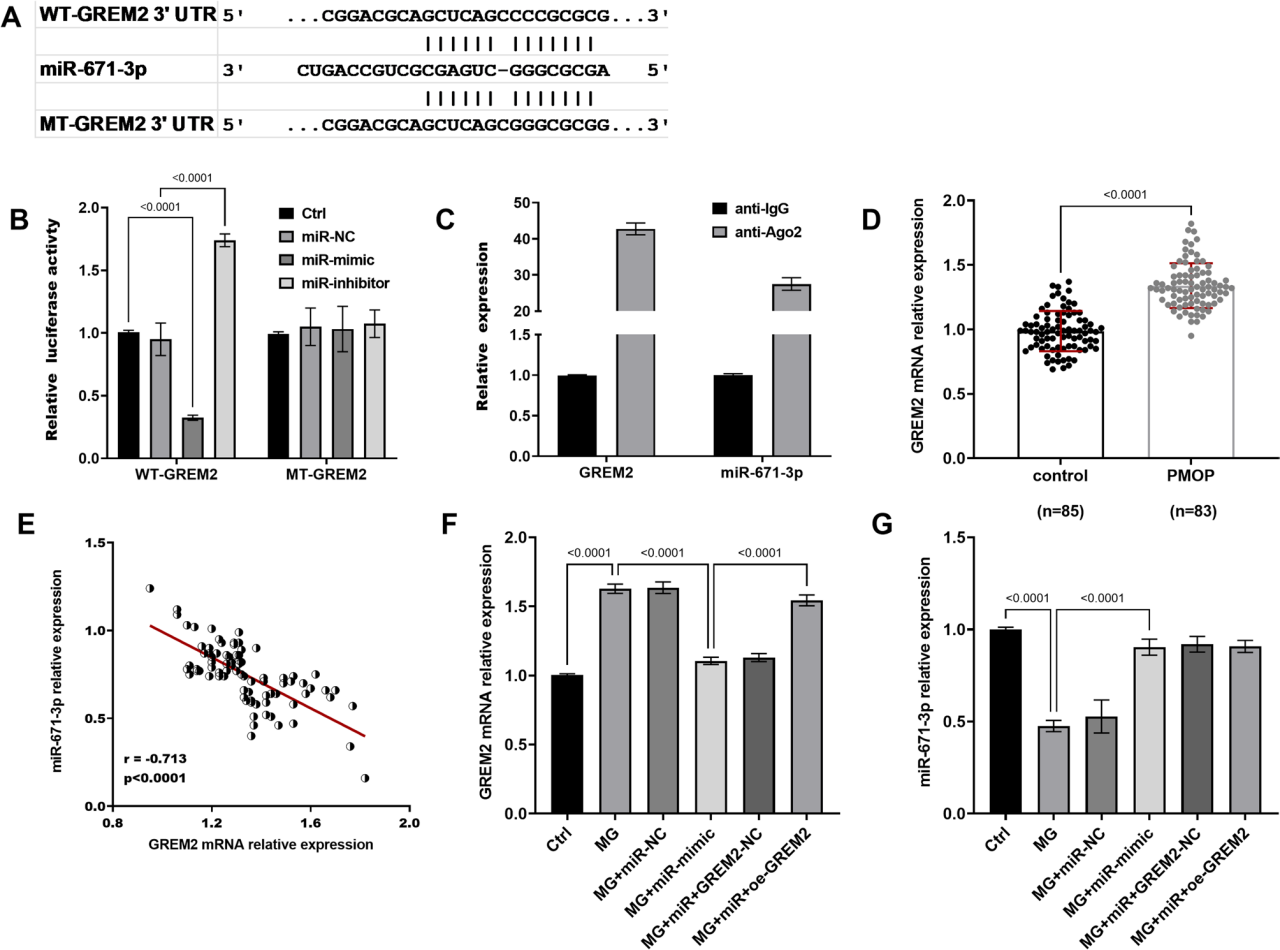
WT/MT-*GREM2* 3’UTR sequences and miR-671-3p binding sites (**A**). Dual-luciferase reporter gene assay (**B**) and RNA immunoprecipitation (RIP) experiment (**C**) indicated the binding between miR-671-3p and *GREM2*. The expression of *GREM2* was upregulated in PMOP patients’ serum (**D**) and negatively related to the levels of miR-671-3p (**E**). *GREM2* was upregulated in MG, and overexpression of miR-671-3p inhibited its expression (**F**). *GREM2* overexpression had no impact on miR-671-3p expression (**G**). WT-*GREM2*: Wild-type *GREM2* 3’UTR luciferase reporter; MT-*GREM2*: Mutant *GREM2* 3’UTR luciferase reporter; Ctrl: Normal culture conditions; MG: Cells treated with simulated MG for 72 h; MG + miR-NC: MG + miR- NC transfection; MG + miR-mimic: MG + miR-671-3p mimic transfection; MG + miR + GREM2-NC: MG + miR-mimic + empty vector transfection; MG + miR + oe-GREM2: MG + miR-mimic + GREM2 overexpression plasmid transfection. Bar graphs show mean ± SD (*n* = 5). two-tailed t-test for WT vs. MT; one-way ANOVA with Tukey’s test for other groups

The binding sites of miR-671-3p within the WT and MT-*GREM2* 3’UTR sequences used for luciferase reporter assays are explicitly illustrated in Fig. 3A. Experimental results demonstrate that alterations in miR-671-3p levels, either up or down, correspondingly decreased or increased the luciferase activity in vectors harboring WT-*GREM2*, while having no influence on vectors with MT-*GREM2* (Fig. 3B). Ago2, a core component of the RISC, mediates miRNA-guided post-transcriptional gene silencing by directly binding to mature miRNAs. RIP assays revealed specific co-enrichment of miR-671-3p and *GREM2* mRNA in Ago2 immunoprecipitates compared to IgG isotype controls (Fig. 3C), confirming their physical interaction within the RNA-induced RISC. Further studies showed that *GREM2* expression was elevated in the serum of patients with PMOP (Fig. 3D), and an inverse relationship was observed between *GREM2* and miR-671-3p expression levels (Fig. 3E, *R* = -0.713, *P* < 0.001). Similarly, *GREM2* expression was upregulated in MC3T3-E1 cells subjected to MG, while miR-671-3p overexpression mitigated this upregulation (Fig. 3F). Conversely, variations in *GREM2* expression did not significantly impact miR-671-3p levels (Fig. 3G).

### Influence of *GREM2* on MC3T3-E1 cell activity in microgravity conditions

**Fig. 4:**
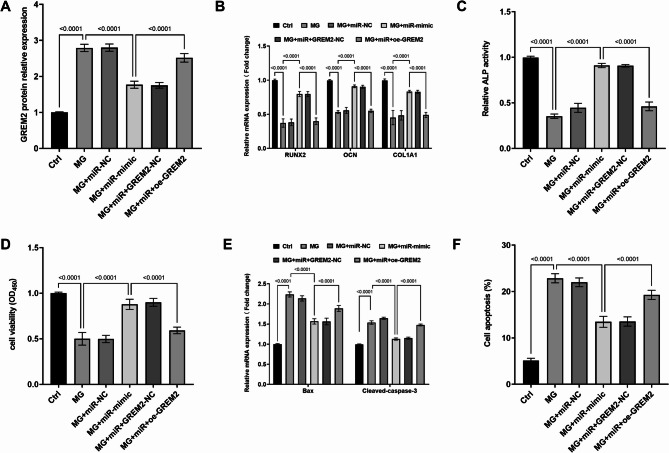
miR-671-3p inhibits *GREM2* protein expression (**A**). Elevated *GREM2* exacerbates MG-induced inhibition of cell differentiation (**B-C**), disrupted viability (**D**) and apoptosis (**E-F**), counteracting the protective effect of miR-671-3p. Ctrl: Normal culture conditions; MG: Cells treated with simulated MG for 72 h; MG + miR-NC: MG + miR- NC transfection; MG + miR-mimic: MG + miR-671-3p mimic transfection; MG + miR + GREM2-NC: MG + miR-mimic + empty vector transfection; MG + miR + oe-GREM2: MG + miR-mimic + GREM2 overexpression plasmid transfection. Bar graphs show mean ± SD (*n* = 5). one-way ANOVA with Tukey’s post hoc test

miR-671-3p mediated targeted downregulation of *GREM2* protein levels, while functional validation through *GREM2* overexpression (oe-GREM2) effectively reversed this suppression (quantified in Figure. 4 A), as evidenced by Western blotting (representative raw images in Fig. S1). Elevated *GREM2* levels intensified the differentiation (Fig. 4B-C) and viability (Fig. 4D) disturbances in MC3T3-E1 cells induced by MG, further accelerating cell apoptosis (Fig. 4E-F) and negating the protective effects of miR-671-3p on MG-exposed MC3T3-E1 cells.

### Modulation of the BMP2/Smad/Akt signaling pathway by miR-671-3p targeting *GREM2*

**Fig. 5:**
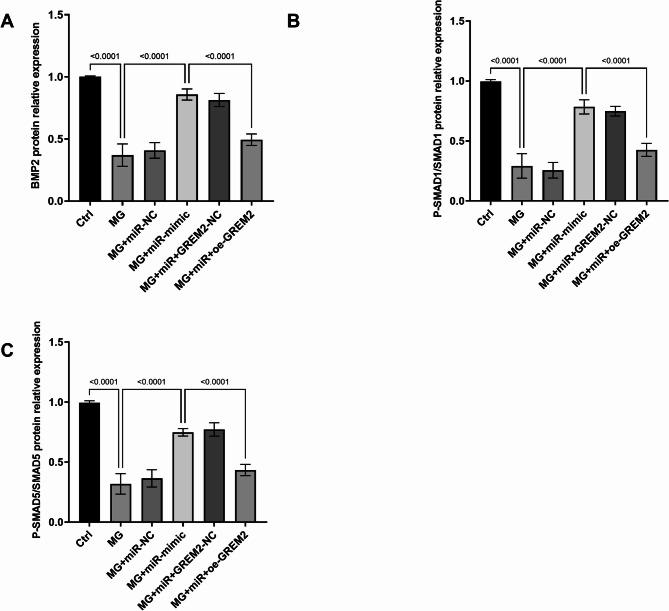
Reduced expression of *BMP2* (**A**) and *P-SMAD1/5* (**B-C**) proteins under MG is promoted by miR-671-3p upregulation and inhibited by *GREM2* overexpression. Ctrl: Normal culture conditions; MG: Cells treated with simulated MG for 72 h; MG + miR-NC: MG + miR- NC transfection; MG + miR-mimic: MG + miR-671-3p mimic transfection; MG + miR + GREM2-NC: MG + miR-mimic + empty vector transfection; MG + miR + oe-GREM2: MG + miR-mimic + GREM2 overexpression plasmid transfection. Bar graphs show mean ± SD (*n* = 5). one-way ANOVA with Tukey’s post hoc test

Given *GREM2*’s known antagonism of *BMP2* signaling and its upregulation under MG conditions, it was hypothesized that miR-671-3p-mediated suppression of *GREM2* would activate the *BMP2/SMAD* pathway, a key driver of osteogenesis. To validate this, it was analyzed the expression of BMP2 and phosphorylated *SMAD1/5* proteins in MG-treated cells. MG significantly reduced*BMP2* protein (Fig. 5A) and phosphorylation of *SMAD1/5* (Fig. 5B-C). However, the upregulation of miR-671-3p overexpression under MG conditions restored expression of both *BMP2* and *p-SMAD1/5*, demonstrating its regulatory capacity. Conversely, overexpression of *GREM2* abrogated these effects, as confirmed by Western blot quantification (Figure S2).

## Discussion

Osteoporotic fractures cause a substantial decline in patients’ quality of life and are closely tied to heightened risks of premature mortality, disability, and financial strain. Therefore, identifying individuals at high risk for these fractures and providing them with appropriate treatment is vital [18]. While Dual-energy X-ray absorptiometry (DXA) for BMD detection is a standard diagnostic method for osteoporosis, it may sometimes result in a delayed diagnosis [19, 20]. In contrast, serum miRNAs hold significant clinical potential due to their direct interaction with bone metabolism markers, long-term storage stability, and capacity to provide timely insights into bone health [21]. Current research focuses on discovering more specific and sensitive markers of bone turnover. Studies have shown that serum miR-636 possesses high sensitivity and specificity in distinguishing whether postmenopausal patients have osteoporosis and may regulate osteoblast activity [22]. Abnormal expression of serum miR-338 has also been linked to the occurrence of PMOP [23]. miR-206 has emerged as a novel potential diagnostic biomarker for osteoporosis [24], and serum miR-578 has been associated with the risk of fragility fractures in PMOP patients [25]. In our study, we found that the serum expression of miR-671-3p was markedly downregulated in PMOP patients. In addition, miR-671-3p demonstrated high specificity and sensitivity in predicting the occurrence of PMOP and was closely correlated with BMD. It was hypothesized that serum miR-671-3p may be a promising target for early prediction of PMOP and is highly associated with the onset of osteoporosis in patients.

Osteoblast differentiation is essential for maintaining the stability and health of bone tissue [26]. To simulate the osteoporotic environment, researchers frequently employ MG treatment of osteoblasts. In microgravity environments, osteoblast differentiation encounters significant challenges and disruptions, which may result in abnormal differentiation and impaired functionality [27, 28]. The MG osteoblast model established in this study further confirmed this. Additionally, MG treatment was found to significantly decrease the expression of miR-671-3p in MC3T3-E1 cells. Upregulation of miR-671-3p notably enhanced the expression of osteogenic markers such as *OCN*, *RUNX2*, *COL1A1*, and ALP activity. *RUNX2* serves as a pivotal switch for osteoblast differentiation [29], *OCN* plays a vital role in regulating the deposition of mineralized crystals [30], *COL1A1* encodes the primary type I collagen crucial for bone development [31]. ALP provides the necessary phosphate for hydroxyapatite deposition, thereby promoting osteogenesis [32]. These observations suggest that miR-671-3p promotes osteoblast differentiation. Furthermore, miR-671-3p was observed to augment osteoblast viability and inhibit apoptosis. Previous studies by Liu et al. indicated that miR-671-3p can regulate chondrocyte apoptosis, proliferation and inflammatory responses by targeting TNF receptor-associated factor 3 (*TRAF3*) [10]. miR-671-3p has also been implicated in regulating proliferation and apoptosis of breast cancer cells [33]. These findings further emphasize the critical role of miR-671-3p in regulating cellular activities. Therefore, it is proposed that miR-671-3p may contribute to the development of osteoporosis by modulating osteoblast activities.

In the current study, *GREM2* was identified as a direct target of miR-671-3p through a systematic selection process. Initial bioinformatics screening using TargetScan and StarBase revealed multiple potential binding candidates for miR-671-3p. Among these, *GREM2* was prioritized for experimental validation due to its predicted high binding affinity and prior evidence linking it to bone metabolism. Dual-luciferase reporter assays confirmed the direct interaction between miR-671-3p and the 3’-UTR of *GREM2*. Furthermore, *GREM2*, a crucial member of the transforming growth factor β (*TGFβ*) superfamily, plays a pivotal role in maintaining bone homeostasis and regulating the equilibrium between bone formation and resorption [34]. Prior research, including Zhou et al., has demonstrated that miR-127-5p can modulate chondrocyte differentiation by targeting *GREM2* [35]. Additionally, studies have shown that the functional loss of *GREM2* stimulates the expression of alkaline phosphatase *ALP* and *OCN* in osteoblasts, thereby promoting osteoblast differentiation and proliferation, ultimately resulting in increased BMD in female rats [36]. These findings align with the results of the study, suggesting that miR-671-3p may regulate osteoblast activities through targeting *GREM2*.

The *BMP/SAMD* pathway was selected for investigation based on its well-established role in regulatingosteoblast differentiation and bone formation. In PMOP, estrogen deficiency disrupts the balance between bone resorption and formation, and *BMP2* is a key growth factor that promotes osteogenesis by activating *SMAD1/5 phosphorylation* [37]. Previous studies have demonstrated that *BMP2/SMAD* signaling is suppressed in osteoporotic models, and its reactivation can rescue bone loss [38]. Notably, *GREM2*, the target of miR-671-3p identified in our study, is a known antagonist of *BMP2*, effectively blocking the BMP signaling pathway [39]. By binding to *BMP2*, *GREM2* blocks receptor interaction and inhibitsdownstream *SMAD* signaling [40]. Based on these results, it is speculated that miR-671-3p targets GREM2 to reduce its inhibition of *BMP2/SMAD* signaling, thereby promoting osteoblast differentiation and contributing to PMOP pathogenesis. This rationale aligns with our experimental findings showing that miR-671-3p overexpression restored *BMP2* and *p-SMAD1/5* levels under microgravity conditions.

This study has several limitations. The limited sample size and homogeneity of the clinical cohort may constrain the generalizability of miR-671-3p’s diagnostic potential across diverse populations [2]. While the simulated microgravity model using murine pre-osteoblasts (MC3T3-E1 cells) offers mechanistic insights, interspecies differences in miRNA regulation and human osteoblast-specific responses remain unverified [3]. In vitro systems fail to replicate the complexity of in vivo bone remodeling, particularly osteoblast-osteoclast crosstalk and systemic hormonal influences in PMOP [4]. Focusing on miR-671-3p and the *BMP2/SMAD* pathway may overlook contributions from other miRNAs or pathways.

Future directions should prioritize: (1) expand clinical cohorts to include multi-ethnic populations and subgroups based on osteoporosis severity; (2) validate findings in human primary osteoblasts or additional cell lines to enhance physiological relevance; (3) utilize co-culture systems (osteoblasts/osteoclasts) and ovariectomized rodent models to assess bone density changes and pathway interactions in vivo; (4) apply multi-omics approaches to identify synergistic regulatory networks; and (5) develop targeted delivery systems to enhance miR-671-3p stability and osteogenic specificity for translational applications. It is emphasized here that while our findings lay the groundwork for clinical translation, the therapeutic potential of miR-671-3p requires rigorous validation in preclinical models to ensure safety and efficacy in human PMOP. In summary, decreased expression of serum miR-671-3p serves as an early indicator for the development of PMOP. miR-671-3p may directly regulate the expression of *GREM2*, subsequently activating the *BMP2/SMAD* signaling pathway. This activation accelerates osteoblast differentiation, promotes bone matrix synthesis and deposition, and enhances bone formation activities at the molecular level, effectively delaying the pathological progression of osteoporosis.

## Electronic supplementary material

Below is the link to the electronic supplementary material.

Supplementary Material 1

Supplementary Material 2

## Data Availability

No datasets were generated or analysed during the current study.

## Abbreviations

BMP2: Bone morphogenetic protein 2
FBS: Fetal bovine serum
IGF2: Insulin-like growth factor 2
MiRNAs: MicroRNAs
PMOP: Postmenopausal osteoporosis
GREM2: Gremlin-2
RUNX2: Runt-related transcription factor 2
OCN: Osteocalcin
COL1A1: Ccollagen, type I, alpha 1
P-SMAD1: Phosphorylated Drosophila Mothers Against Decapentaplegic Protein 1
PVDF: Polyvinylidene difluoride
TBST: Tris-Buffered Saline with Tween-20
ECL: Enhanced Chemiluminescence
ROC: Receiver Operating Characteristic
BMD: Bone mineral density
LS: Lumbar spine
FN: Femoral neck
TH: Total hip
DXA: Dual-energy X-ray absorptiometry
TGFβ: Transforming growth factorβ
OVX: Ovariectomized
PP2A: Protein phosphatase 2 A
