# Supplementary figures and images for: Overexpression of miR-671-3p alleviates postmenopausal osteoporosis by targeting GREM2 to activate BMP2/SMAD signaling pathway
Source: Hereditas. 2025 Jun 11;162:102. doi: 10.1186/s41065-025-00467-8 (PMC12153117; doi:10.1186/s41065-025-00467-8)

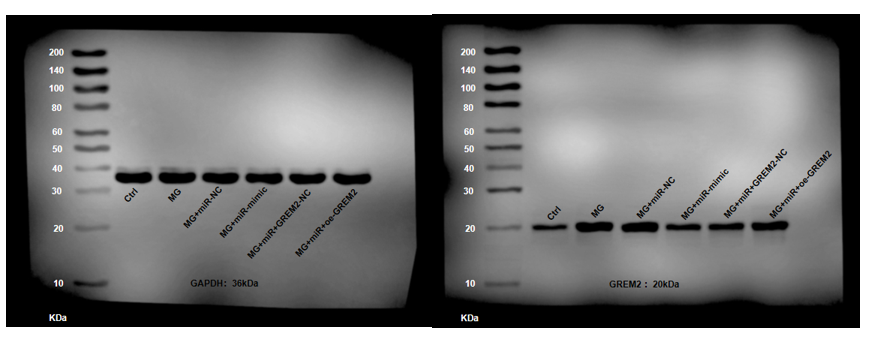

Supplement: Supplementary file 1 — Supplementary Material 1 [file 41065_2025_467_MOESM1_ESM.tif]

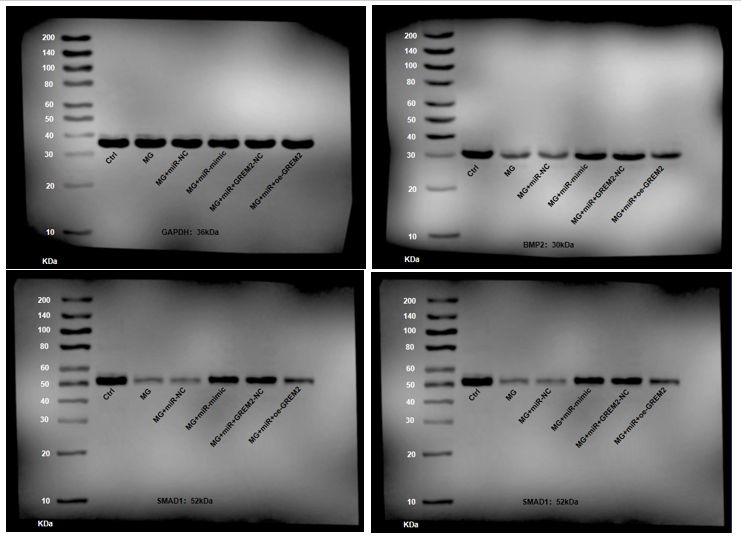

Supplement: Supplementary file 2 — Supplementary Material 2 [file 41065_2025_467_MOESM2_ESM.tif]
